# Coccidioidal Meningitis in a Patient With HIV

**DOI:** 10.7759/cureus.76001

**Published:** 2024-12-19

**Authors:** Patrick M Byrne, Sanjay Shrestha

**Affiliations:** 1 Family Medicine Alexandria, Louisiana State University Health Sciences Center, Alexandria, USA

**Keywords:** aids (acquired immunodeficiency syndrome), coccidioidal meningitis, coccidiomycosis, hiv diseases, invasive fungal infections

## Abstract

Coccidioidal meningitis is a rare yet life-threatening complication of disseminated *Coccidioides *infection, primarily affecting immunocompromised individuals. *Coccidioides immitis* and *Coccidioides posadasii* are dimorphic fungi endemic to the southwestern United States, where exposure to inhaled spores can lead to a range of clinical manifestations, including pulmonary and central nervous system (CNS) involvement. This report describes the case of a 27-year-old correctional officer with advanced human immunodeficiency virus (HIV) infection who presented with chronic headaches, altered mental status, and significant weight loss following his relocation from Arizona to Louisiana. Cerebrospinal fluid (CSF) analysis revealed slow-growing mold consistent with *Coccidioides* species, leading to the diagnosis of coccidioidal meningitis. This case emphasizes the importance of considering fungal meningitis in patients with compatible geographic exposure and immunosuppression.

## Introduction

Coccidioidal meningitis (CM) is a severe and potentially fatal central nervous system (CNS) manifestation of disseminated *Coccidioides* infection. The causative fungi, *Coccidioides immitis* and *Coccidioides posadasii*, are endemic to the southwestern United States, including Arizona, California, Nevada, and New Mexico [1]. Infection occurs following inhalation of airborne arthroconidia from desert soil, which can lead to pulmonary disease. While the majority of infections are self-limited, dissemination occurs in a small subset of patients, particularly those who are immunocompromised, such as individuals with untreated human immunodeficiency virus (HIV) and acquired immunodeficiency syndrome (AIDS) [2-3].

CM accounts for approximately 30%-50% of disseminated coccidioidomycosis cases and remains one of the most serious complications due to its chronic, progressive nature and the risk of devastating neurological outcomes [4]. Symptoms often develop gradually, and the disease can be difficult to diagnose, as the fungi grow slowly in culture and symptoms overlap with other causes of meningitis [2]. Prompt recognition and treatment with antifungal therapy are critical for survival and reducing complications [5].

Here, we present the case of a 27-year-old male correctional officer with advanced HIV who relocated from Arizona, a region endemic for *Coccidioides*, to Louisiana. The patient presented with classic symptoms of chronic meningitis, and cerebrospinal fluid (CSF) studies confirmed the diagnosis of CM. This case highlights the importance of geographic history, the role of immunosuppression in disease dissemination, and the need for early diagnosis to improve outcomes [1-3].

## Case presentation

A 27-year-old male correctional officer presented to the emergency department with a two-week history of progressively worsening headaches, confusion, fatigue, and unintentional weight loss. The patient had recently relocated from Arizona to Louisiana for employment, where he worked outdoors in a high-exposure environment as a prison guard. He described the headaches as constant, global, and non-throbbing, with occasional neck stiffness. Over the past week, his family reported episodes of intermittent confusion, fevers, irritability, and difficulty concentrating. He denied seizures, focal weakness, vision changes, or photophobia but endorsed nausea and decreased appetite.

The patient's medical history was significant for HIV, but he was unsure of his current CD4 count. The patient had been prescribed bictegravir/emtricitabine/tenofovir alafenamide (Biktarvy) but reported non-compliance with the antiretroviral medication due to cost. He had no history of opportunistic infections and was diagnosed with HIV four years before his presentation. Social history revealed that he was a non-smoker, and he denied alcohol or drug use. He lived alone and had no pets. There was no recent travel history outside Arizona and Louisiana, but he worked in an outdoor setting in a prison yard in Arizona.

On examination, the patient was alert but appeared fatigued and moderately disoriented. Vital signs revealed a low-grade fever of 100.8°F (38.2°C), tachycardia at 110 beats per minute, a respiratory rate of 20 breaths per minute, a blood pressure of 134/74 mm/Hg, and an oxygen saturation of 98% in room air as read by pulse oximetry (Table 1).

**Table 1:** Initial vital signs in the emergency department

Temperature	Heart rate	Respiratory rate	Blood pressure	Oxygen saturation
100.8°F	110 beats per minute	20 breaths per minute	134/74 mmHg	98% oxygen saturation in room air

He exhibited mild neck stiffness, with positive Kernig’s (pain and resistance on extending the knee with the hip flexed) and Brudzinski’s (involuntary hip and knee flexion with passive neck flexion) signs, consistent with meningeal irritation. Cranial nerve examination was normal, and there were no focal neurological deficits. Cardiopulmonary and abdominal examinations were unremarkable. Skin inspection revealed no rashes, lesions, or signs of disseminated fungal disease.

Laboratory studies showed mild hyponatremia and normal blood counts and metabolic panel. The patient’s CD4 count was 49 cells/mm³, confirming significant immunosuppression (Table 2).

**Table 2:** Initial laboratory evaluation including a complete blood count (CBC) and complete metabolic panel (CMP) GFR: glomerular filtration rate

Test	Value	Reference range
Sodium	130 mmol/L	135-148 mmol/L
Potassium	3.9 mmol/L	3.3-5.1 mmol/L
Chloride	95 mmol/L	98-107 mmol/L
Carbon dioxide (CO_2_)	23 mmol/L	21-32 mmol/L
Anion gap	12	8-15
Blood urea nitrogen (BUN)	12 mg/dL	6-19 mg/dL
Creatinine	0.93 mg/dL	0.70-1.30 mg/dL
Estimated GFR	>90 mL/min	>90 mL/min
Glucose	124 mg/dL	70-120 mg/dL
Calculated osmolality	271 mOsm/kg	275-305 mOsm/kg
Calcium	9.8 mg/dL	8.4-10.2 mg/dL
Corrected calcium	9.7 mg/dL	8.4-10.2 mg/dL
Total bilirubin	0.7 mg/dL	0.1-1.0 mg/dL
Aspartate aminotransferase (AST)	19 U/L	0-37 U/L
Alanine transaminase (ALT)	25 U/L	0-40 U/L
Total alkaline phosphatase (ALP)	70 U/L	40-130 U/L
White blood cells (WBCs)	7.7 cells/µL	4.5-11.0 cells/µL
Red blood cells (RBCs)	5.03 million cells/µL	4.7-6.1 million cells/µL
Hemoglobin (Hgb)	15.0 g/dL	13.5-17.5 g/dL
Hematocrit (Hct)	43.3%	40%-54%
Mean corpuscular volume (MCV)	86.1 fL	80-100 fL
Mean corpuscular hemoglobin (MCH)	29.8 pg/cell	27-33 pg/cell
Mean corpuscular hemoglobin concentration (MCHC)	34.6 g/dL	32-36 g/dL
Red cell distribution width (RDW)	11.9%	11-15%
Platelet count	335.0 platelets/µL	150-450 platelets/µL
Mean platelet volume (MPV)	8.9 fL	7.4-10.4 fL

A lumbar puncture was performed, with CSF analysis revealing an elevated opening pressure of 28 cm H₂O, a lymphocytic pleocytosis (79 white blood cells (WBCs)/mm³), elevated protein (116.5 mg/dL), and low glucose (28 mg/dL) (Table 3).

**Table 3:** Cerebrospinal fluid (CSF) laboratory analysis WBC: white blood cell; RBC: red blood cell

Test	Value	Reference range
CSF opening pressure	28 cm H_2_O	6-20 cm H_2_O
CSF glucose	28 mg/dL	40-70 mg/dL
CSF total protein	116.5 mg/dL	15-45 mg/dL
CSF beta-2-microglobulin	4.2 mg/dL	<2.0 mg/dL
CSF WBC	79 cells/µL	0-5 cells/µL
CSF RBC	151 cells/µL	0 cells/µL
CSF neutrophils	36%	0%-6%
CSF lymphocytes	64%	40%-80%
CSF mesothelial cells	3	0-3
CSF appearance	Clear	Clear
CSF color	Colorless	Colorless
CSF culture	Mold	Negative

**Figure 1:**
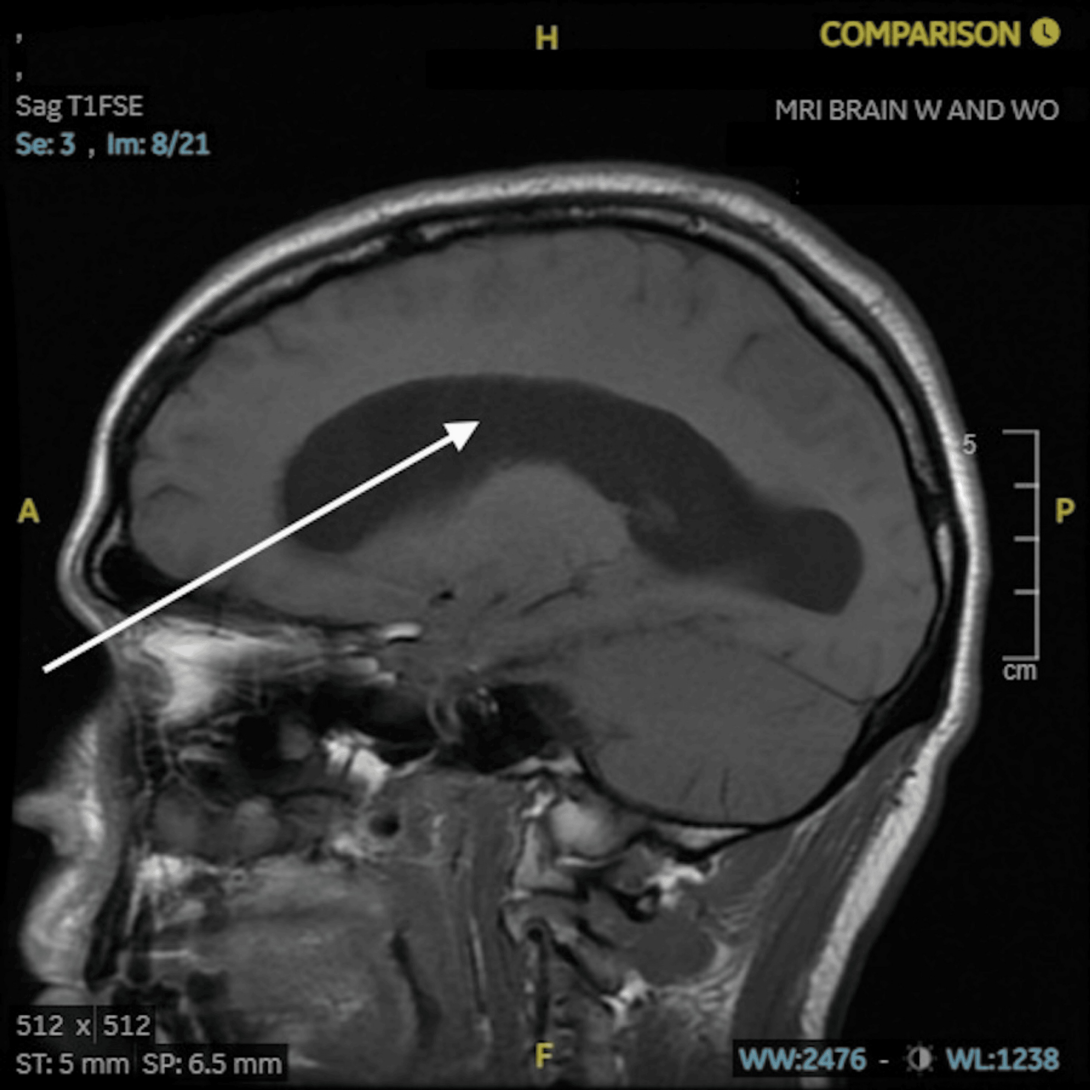
MRI without contrast sagittal view T1-weighted, demonstrating dilated ventricles and hydrocephalus MRI: magnetic resonance imaging

CSF cultures showed slow-growing mold in two vials, which were eventually confirmed to be *Coccidioides*, and polymerase chain reaction (PCR) testing confirmed the presence of *Coccidioides* DNA. Serum serology was positive for *Coccidioides* immunoglobulin G (IgG) antibodies, further supporting the diagnosis. The initial magnetic resonance imaging (MRI) of the head showed significant hydrocephalus in the emergency department (Figure 1).

**Figure 2:**
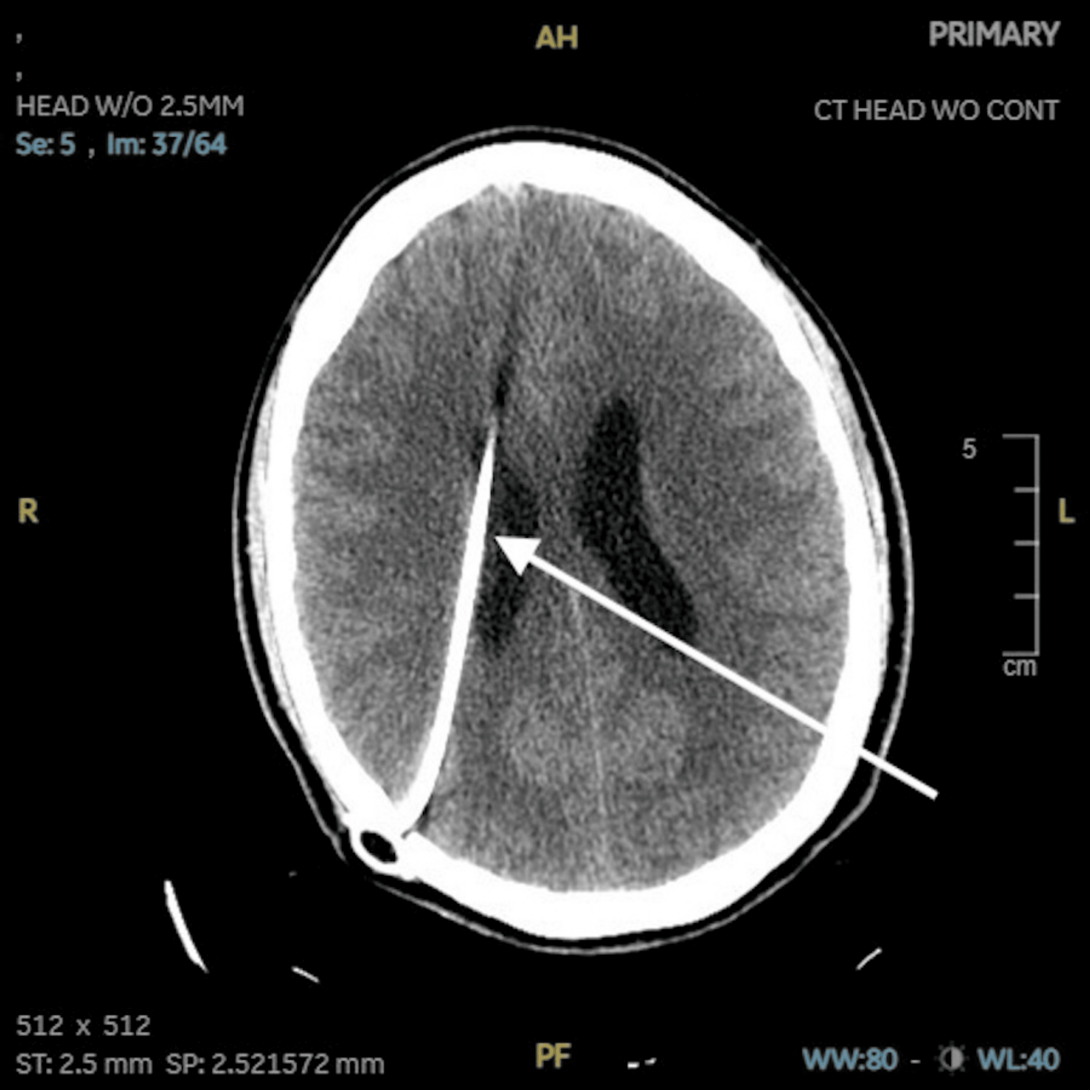
CT scan of the head axial view demonstrating a VP shunt and improved ventricular dilation and hydrocephalus CT: computed tomography; VP: ventriculoperitoneal

Neurosurgery was consulted, and a ventriculoperitoneal (VP) shunt was placed with almost complete resolution of disorientation and somnolence and improvement in ventricular dilation on a subsequent computed tomography (CT) scan (Figure 2).

**Figure 3:**
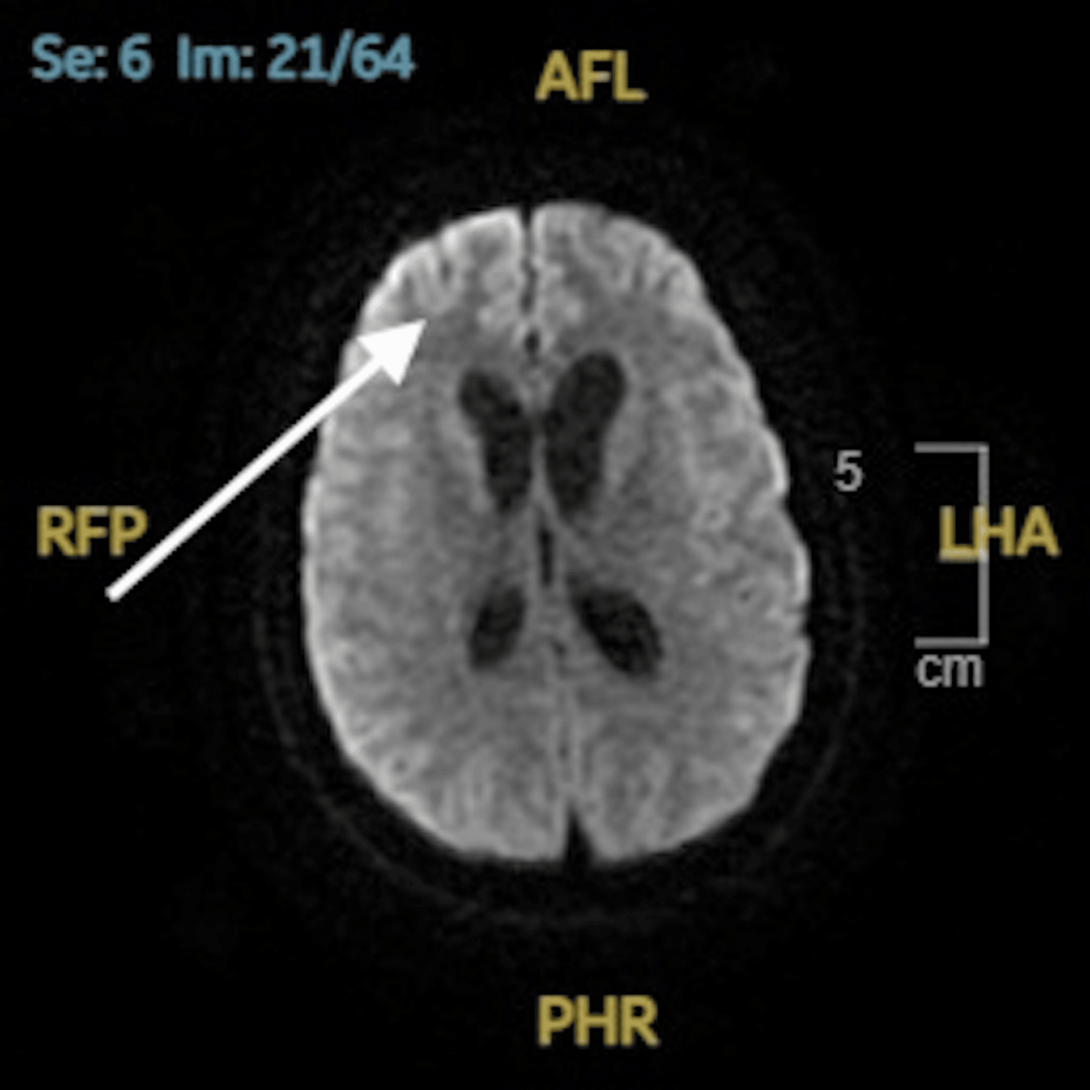
MRI brain axial view with contrast diffusion-weighted demonstrating leptomeningeal enhancement suggestive of meningitis MRI: magnetic resonance imaging; AFL: anterior left; LHA: left anterior; PHR: posterior right; RFP: right posterior

A follow-up brain MRI demonstrated mild leptomeningeal enhancement, suggesting meningitis without evidence of infarction or abscess formation (Figure 3).

**Figure 4:**
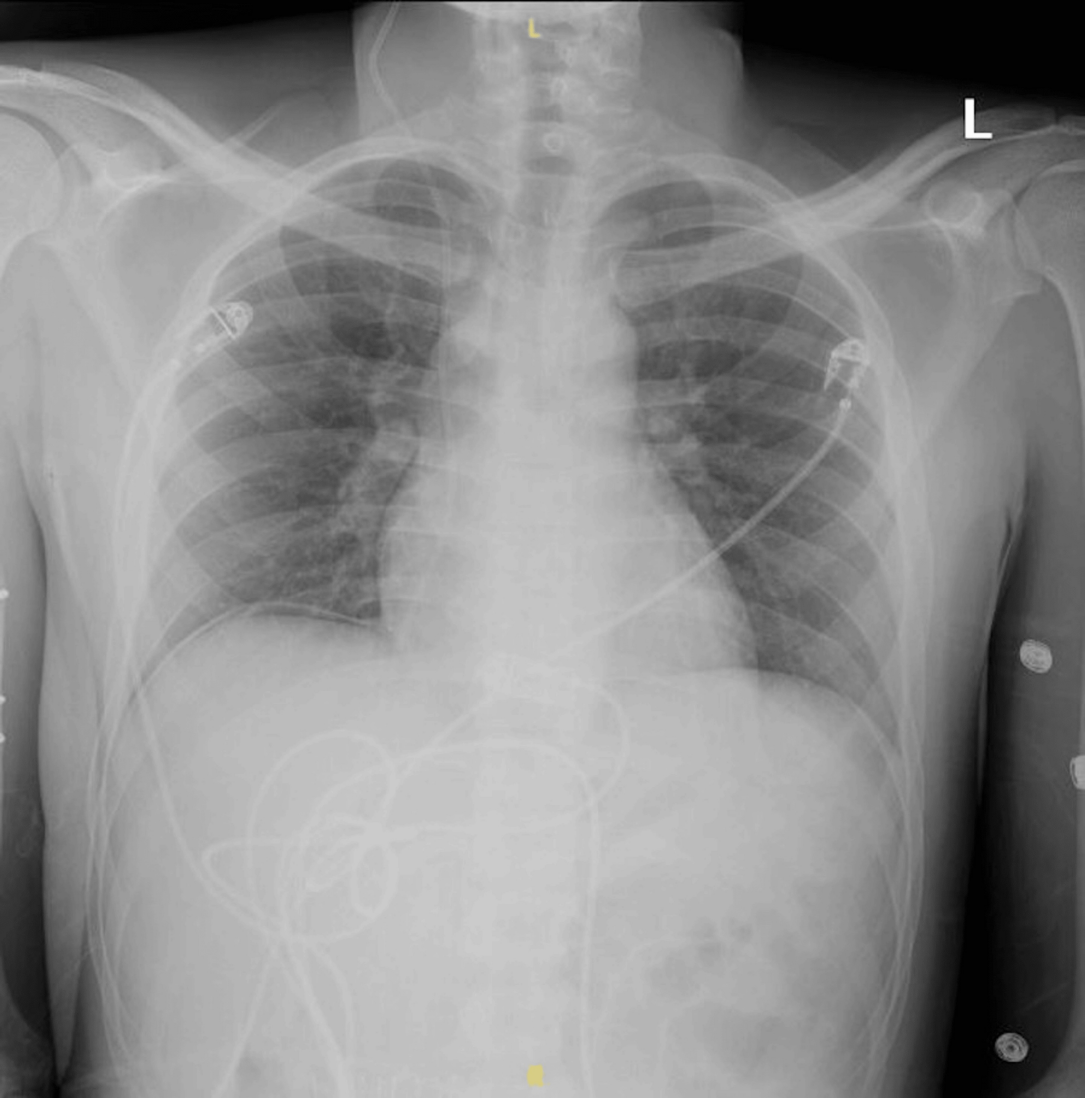
Chest radiography without any evidence of pulmonary coccidiosis

A chest radiograph was performed to assess for pulmonary involvement, and no abnormal findings were identified (Figure 4).

Based on the clinical presentation, geographic history, CSF findings, and imaging, the patient was diagnosed with CM and was started on 800 mg of oral fluconazole (Diflucan) therapy. The patient was instructed to continue the therapy for life. Consultations with infectious disease and neurology specialists were obtained to guide management.

## Discussion

CM represents one of the most serious and life-threatening complications of disseminated *Coccidioides *infection. *C. immitis* and *C. posadasii* are dimorphic fungi endemic to the arid regions of the southwestern United States, particularly Arizona, California, Nevada, and New Mexico, where they exist as arthroconidia in desert soil [1-2]. Infection occurs primarily through inhalation of airborne arthroconidia following soil disruption, a risk heightened by occupational exposures such as construction, agriculture, or outdoor work. In the United States, Arizona accounts for the majority of reported infections, with rates as high as 150 cases per 100,000 people annually in endemic areas [3]. Immunosuppressed patients, including those with untreated or advanced HIV, have a significantly increased susceptibility to disseminated disease, with CNS involvement occurring in up to 30%-50% of disseminated cases [4].

In this case, the patient’s geographic history, combined with his advanced HIV status and CD4 count of 49 cells/mm³, placed him at particularly high risk for disseminated *Coccidioides *infection. Hematogenous dissemination to the meninges leads to chronic inflammation characterized by lymphocytic pleocytosis, elevated CSF protein, and decreased CSF glucose, as seen in this case. If left untreated, meningeal fibrosis can result in hydrocephalus, vasculitis, and infarction, leading to progressive neurological decline [2,4]. The patient presented with a classic subacute course, including progressive headaches, neck stiffness, confusion, and weight loss. These symptoms align with the typical presentation of CM, which often progresses insidiously over weeks to months [3,5]. On physical examination, positive Kernig's and Brudzinski's signs were observed, consistent with meningeal irritation in fungal meningitis.

CSF analysis was diagnostic, revealing elevated opening pressure, a lymphocytic predominance (79 WBCs/mm³), elevated protein (116.5 mg/dL), and hypoglycorrhachia (28 mg/dL), which are hallmarks of fungal meningitis [5]. The slow-growing mold identified on CSF culture was later confirmed as *Coccidioides* via PCR testing. PCR testing has become invaluable in expediting diagnosis, as fungal cultures may take up to seven to 14 days to confirm the presence of *Coccidioides* [6]. Positive serology for coccidioidal IgG antibodies in the serum further supported the diagnosis. Imaging studies, including brain MRI, demonstrated mild leptomeningeal enhancement without evidence of abscess or infarction, findings consistent with early-stage CM [4,6]. Notably, the patient’s chest radiography was normal, indicating no pulmonary involvement at the time of diagnosis. However, pulmonary disease is commonly seen in disseminated cases and may demonstrate consolidations, effusions, cavitary lesions, or nodules on chest radiography [4-6].

Prompt diagnosis and initiation of antifungal therapy are critical for improving outcomes in patients with CM. High-dose oral fluconazole (400-800 mg/day) is the standard first-line treatment due to its excellent CNS penetration and favorable side effect profile [7]. Lifelong suppressive therapy with treatment-dose fluconazole is often required to prevent relapse, as the disease is chronic and relapsing, particularly in immunocompromised hosts. In severe or refractory cases, intrathecal or intravenous amphotericin B may be required, though its use is associated with significant toxicity [2,5,7]. For this patient, fluconazole was initiated, and consultations with infectious disease and neurology specialists ensured appropriate management and monitoring. The decision of when to initiate antiretroviral therapy (ART) may be challenging. It is not required to delay the administration of ART after CM is diagnosed; however, it may be prudent to wait four to eight weeks after antifungal therapy is started before beginning ART to avoid immune reconstitution inflammatory syndrome (IRIS), especially in patients with a low initial CD4 count. In either circumstance, it is essential to have consistent follow-up to monitor the patient's progress, symptoms, and labs [5-7].

Complications of untreated or inadequately managed CM include hydrocephalus, vasculitis, seizures, cranial nerve deficits, and permanent cognitive impairment. Hydrocephalus, in particular, is a common sequela of chronic meningeal inflammation, often necessitating surgical intervention, such as VP shunting [2,4,6]. Early antifungal therapy significantly reduces the risk of these outcomes, though close monitoring remains essential to detect and manage complications promptly.

The differential diagnosis for chronic meningitis includes cryptococcal meningitis, tuberculous meningitis, neurosarcoidosis, and malignancy. Cryptococcal meningitis, which is particularly common in HIV-positive individuals, was initially considered in this case; however, CSF culture and PCR testing ruled out Cryptococcus neoformans and confirmed *Coccidioides* as the causative pathogen [6-7]. Tuberculous meningitis can present similarly but often features higher CSF protein levels and acid-fast bacilli on staining.

The prognosis of CM has improved significantly with the advent of azole antifungal therapy, though lifelong management is often necessary. Mortality without treatment approaches 100%, while survival rates improve markedly with timely intervention and long-term antifungal suppression [2,7]. Preventive measures include reducing exposure to endemic soils, particularly for immunocompromised individuals, by avoiding high-risk activities such as excavation or outdoor work. Additionally, patients with HIV should be maintained on ART to optimize immune function and reduce the risk of disseminated infections [1,3,7].

## Conclusions

This case highlights the importance of considering CM in patients with a compatible travel or occupational history and immunosuppression who present with chronic meningitic symptoms. Early recognition, appropriate CSF analysis, and timely antifungal therapy are critical to preventing complications and improving outcomes. Timely diagnosis relies on CSF analysis, fungal cultures, and advanced molecular techniques, such as PCR, which expedite pathogen identification. CSF findings of elevated opening pressure, lymphocytic pleocytosis, elevated protein, and hypoglycorrhachia, as observed in this case, are critical clues for diagnosing fungal meningitis. Clinicians should maintain a high index of suspicion, particularly in immunocompromised patients, such as those with advanced HIV, as a delayed diagnosis can result in devastating neurological sequelae, including hydrocephalus, vasculitis, and chronic cognitive impairment.
